# Neural networks underlying affective states in a multimodal virtual environment: contributions to boredom

**DOI:** 10.3389/fnhum.2013.00820

**Published:** 2013-11-28

**Authors:** Krystyna A. Mathiak, Martin Klasen, Mikhail Zvyagintsev, René Weber, Klaus Mathiak

**Affiliations:** ^1^Department of Child and Adolescent Psychiatry, Psychosomatics and Psychotherapy, RWTH Aachen UniversityAachen, Germany; ^2^Department of Psychiatry, Psychotherapy and Psychosomatics, RWTH Aachen UniversityAachen, Germany; ^3^Jülich-Aachen Research Alliance (JARA)-Translational Brain MedicineJülich, Germany; ^4^Department of Communication-Media Neuroscience Lab, University of CaliforniaSanta Barbara, CA, USA

**Keywords:** boredom, negative affect, positive affect, video game, PANAS

## Abstract

The interaction of low perceptual stimulation or goal-directed behavior with a negative subjective evaluation may lead to boredom. This contribution to boredom may shed light on its neural correlates, which are poorly characterized so far. A video game served as simulation of free interactive behavior without interruption of the game’s narrative. Thirteen male German volunteers played a first-person shooter game (*Tactical Ops: Assault on Terror*) during functional magnetic resonance imaging (fMRI). Two independent coders performed the time-based analysis of the audio-visual game content. Boredom was operationalized as interaction of prolonged absence of goal-directed behavior with lowered affect in the Positive and Negative Affect Schedule (PANAS). A decrease of positive affect (PA) correlated with response amplitudes in bilateral insular clusters extending into the amygdala to prolonged inactive phases in a game play and an increase in negative affect (NA) was associated with higher responses in bilateral ventromedial prefrontal cortex (vmPFC). Precuneus and hippocampus responses were negatively correlated with changes in NA. We describe for the first time neural contributions to boredom, using a video game as complex virtual environment. Further our study confirmed that PA and NA are separable constructs, reflected by distinct neural patterns. PA may be associated with afferent limbic activity whereas NA with affective control.

## Introduction

Arousal theories define boredom as the state of non-optimal arousal that ensues when there is a mismatch between an individual’s needed arousal and the availability of environmental stimulation (e.g., Csikszentmihalyi, 1975, 1990); it is the aversive state that occurs when it is not possible to achieve an optimal level of arousal through engagement with the environment. Boredom is particularly likely to occur when a task provides little external support for keeping attention engaged, such that performance relies instead on self-sustained attention (Eastwood et al., 2012). Considering video games, this may refer to prolonged situations where the player has no apparent task. Other authors however emphasize aversive aspects of boredom, such as feelings of displeasure, sadness, emptiness, anxiety, and even anger (Csikszentmihalyi, 1975; Csimathkszentmihalyi, 2000; Fahlman et al., 2013).

One aspect of boredom is the interaction of behavior and affect, i.e., reduced affect associated with a lack of goal-oriented behavior. Many researchers suggested that “wishing to, but being unable to, become engrossed in satisfying activity” reflects the state of boredom (for a review, see Eastwood et al., 2012). It is, however, important to remember that task-irrelevant daydreaming or mind wandering is not typically linked with negative mood (Killingsworth and Gilbert, 2010) and rather can be experienced as pleasant engagement (Eastwood et al., 2012). Therefore, the combination of low goal-directed activity with subsequent deterioration of affect is one contribution to boredom.

Boredom is an important and very common phenomenon that, despite its potential significant psychosocial consequences, is still poorly understood (Eastwood et al., 2012). To the best of our knowledge, no study to date has specifically investigated its neural correlates. Virtual environments, particularly video games, can be used as a model to study neuronal processes involved in semi-naturalistic behavior that in a classical block or event-related functional magnetic resonance imaging (fMRI) paradigm would not be accessible (Mathiak and Weber, 2006). We examined neural contributions to boredom using the interactive virtual reality model of a first-person shooter video game and subjective evaluation of affect change due to game play. Since it is not possible to reliably measure the subjective affective state during game play without interrupting it (Klasen et al., 2008, 2011; Weber et al., 2009a,b), we applied the Positive and Negative Affect Schedule (PANAS; Watson et al., 1988) directly before and after the fMRI measurement to measure the affect change due to game playing. Reduced goal-directed behavior may be accompanied by the subjective feeling of boredom and will be reflected in the increase of the negative affect (NA) or a decrease of the positive affect (PA). We expect that the changes in NA and PA should evoke separate activation patterns. The increase of brain activity during prolonged inactivity phases in individuals whose NA is increased or PA is decreased after the game will reflect the subjective feeling of boredom. In addition to emotion processing areas, resting state networks were candidate areas.

## Materials and Methods

### Participants

We recruited 13 male German volunteers (age 18–26, median 23) by means of ads posted at the local university and in video game stores. All participants were right-handed according to the Edinburgh Handedness Inventory (Oldfield, 1971) and considered themselves as regular players of video games (> 5 h/wk, 7–28, median 13 h/wk). Individuals who reported in their history contraindication against magnetic resonance (MR) investigations or neurological, psychiatric or ophthalmologic disorders were excluded from the study. All participants gave their written informed consent and the local ethics committee approved the study protocol.

### Imaging paradigm

After getting acquainted with the game and the controllers for at least 30 min, the volunteers played a violent video game “*Tactical Ops: Assault on Terror”* (Infogrames Europe, Villeurbanne, France) during five functional imaging sessions (except for three participants, who played only four sessions). In the game, the players played freely and experienced the action from the perspective of the virtual character that they control (first-person perspective), while other characters were controlled by the computer. An MR-compatible trackball with five buttons was used by the players to control the game. The participants had time to get acquainted with the controller before the fMRI experiment and the game sound level was adjusted individually (for details, see Weber et al., 2006).

During each 12 min session we recorded hemodynamic brain activity with triple-echo single-shot echo-planar imaging (EPI; repetition time TR = 2.25 s; echo times TE = 23, 40, and 62 ms; 64 × 48 matrix with 4 × 4 mm^2^ resolution; 24 slices with 4 mm thickness plus 1 mm gap; 220 volumes) using a 3T MR scanner (Magnetom Trio, Siemens, Erlangen, Germany). As compared to the conventional single-echo EPI, this technique may increase sensitivity to the blood oxygenation level dependent (BOLD) effect as well as reduce drop-outs and distortions (Weiskopf et al., 2005). We recorded the video display of the game play with the audio track for content analysis. The synchronization with the fMRI data was provided by recording the scanner pulses as second audio track. The fMRI data have been evaluated previously using a different content analysis (see Klasen et al., 2011). We acquired anatomical data from each participant before the functional sessions, for functional coregistration (*T1*-weighted 3d magnetization-prepared rapid acquisition with gradient echo, MPRAGE, 256 × 224 × 160 matrix with 1 mm isotropic voxels).

### Inventories

Participants completed the PANAS (Watson et al., 1988; German version in Krohne et al., 1996) directly before entering and after leaving the MR scanner. The questionnaire contains 20 adjectives describing positive or negative emotions. Each item is rated on a 5-point scale ranging from “very slightly or not at all” to “extremely”, with a total score of 10–50 points per scale.

### Content analysis

Two independent coders and one supervisor performed the time-based analysis of game content at high time resolution (for details, see Weber et al., 2009a,b). Goal oriented behavior can be assumed most of the time course. From a behavioral perspective remarkable phases are prolonged safe situations, with no apparent task. Those phases where the participants have no actual task and do not change it over for more than 10 s, we defined as being absent of goal-directed behavior. The response patterns to the absence of goal-directed behavior were considered for the boredom analysis.

### fMRI data analysis

The reconstructed images underwent artifact reduction: construction of dynamic distortion maps from triple-echo EPI with alternating phase-encoding direction as well as subsequent matching of the three echoes (Weiskopf et al., 2005; Mathiak et al., 2012), a combination of the three echoes weighted with TE^*^S_TE_ based on expected contrast from the averaged signal decay (Mathiak et al., 2004). We conducted statistical parametric mapping following the standard SPM procedures. Preprocessing comprised motion correction and smoothing after normalization into the Montreal Neurological Institute (MNI; Collins et al., 1994) template space of functional and anatomical data; smoothing with 12 mm full-width at half-maximum Gaussian kernel; general linear model constructed from the coding events convoluted with hemodynamic response function as independent variables; and random effect model for group analysis corrected for multiple testing across the entire brain volume (family wise error (FWE) correction; for further details, see Mathiak et al., 2004).

Neuronal networks were disentangled that activate during phases with lack of goal-directed behavior. The BOLD response was modeled by a generic hemodynamic response to these phases. Therefore for each individual, contrast maps were extracted that represented change of neural activity during phases with low goal-oriented behavior. To investigate their relation with affective evaluation the interaction with affect change was evaluated. Therefore, we calculated the inter-subject regression models with the individual change in PA and in NA measures as predictors for the contrast maps. Considering an high inter-individual variability of networks subserving affective evaluation, we applied a cluster corrected threshold, i.e., we considered only clusters with a size larger than a threshold according to *p* < 0.05 corrected for multiple comparisons across the brain volume after applying a voxel-wise threshold according to *p* < 0.01; we previously found these parameters most efficient to detect distributed networks rather than circumscribed areas (Mathiak et al., 2011). Calculations were conducted with statistical parametric mapping software (SPM5, Wellcome Department of Imaging Neuroscience, London, UK) and Matlab 7.1 (The Mathworks Inc., Natick, MA, USA).

## Results

All participants were able to play the game successfully inside the fMRI scanner. The participants scored on average 30.4 ± 4.0 on the positive and 13.0 ± 3.2 on the negative scale of PANAS before the game. After game play, on the scale of PA they reported 26.5 ± 5.1 and on the NA 11.8 ± 3.4, reflecting in general a slight decrease in the intensity of affect (PA: *t*(12) = 2.90, *p* = 0.013; NA: *t*(12) = 1.14, *p* = 0.447). Phases with minimal goal-directed behavior occurred with a frequency of 10.5 ± 3.8 times per 12 min playing session with an average duration of 14.6 ± 15.8 s, resulting in 17.3 ± 9.8% of the recorded playing time.

**Figure 1:**
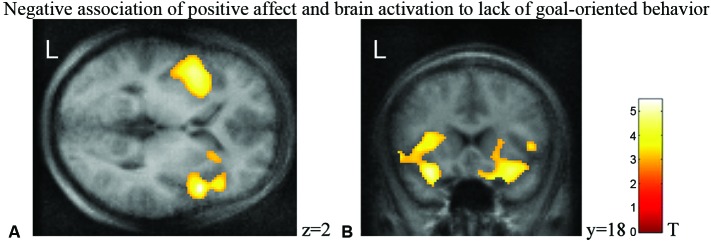
**Statistical maps of behavioral prediction of lower individual responsiveness to lack of goal-oriented behavior (threshold for cluster size according to *p* < 0.05 corrected)**. Bilateral clusters revealed a negative association of brain reactivity to lack of goal-oriented behavior in **(A)** the insula and **(B)** the amygdala. Negative association of PA and brain activation to lack of goal-oriented behavior.

**Figure 2:**
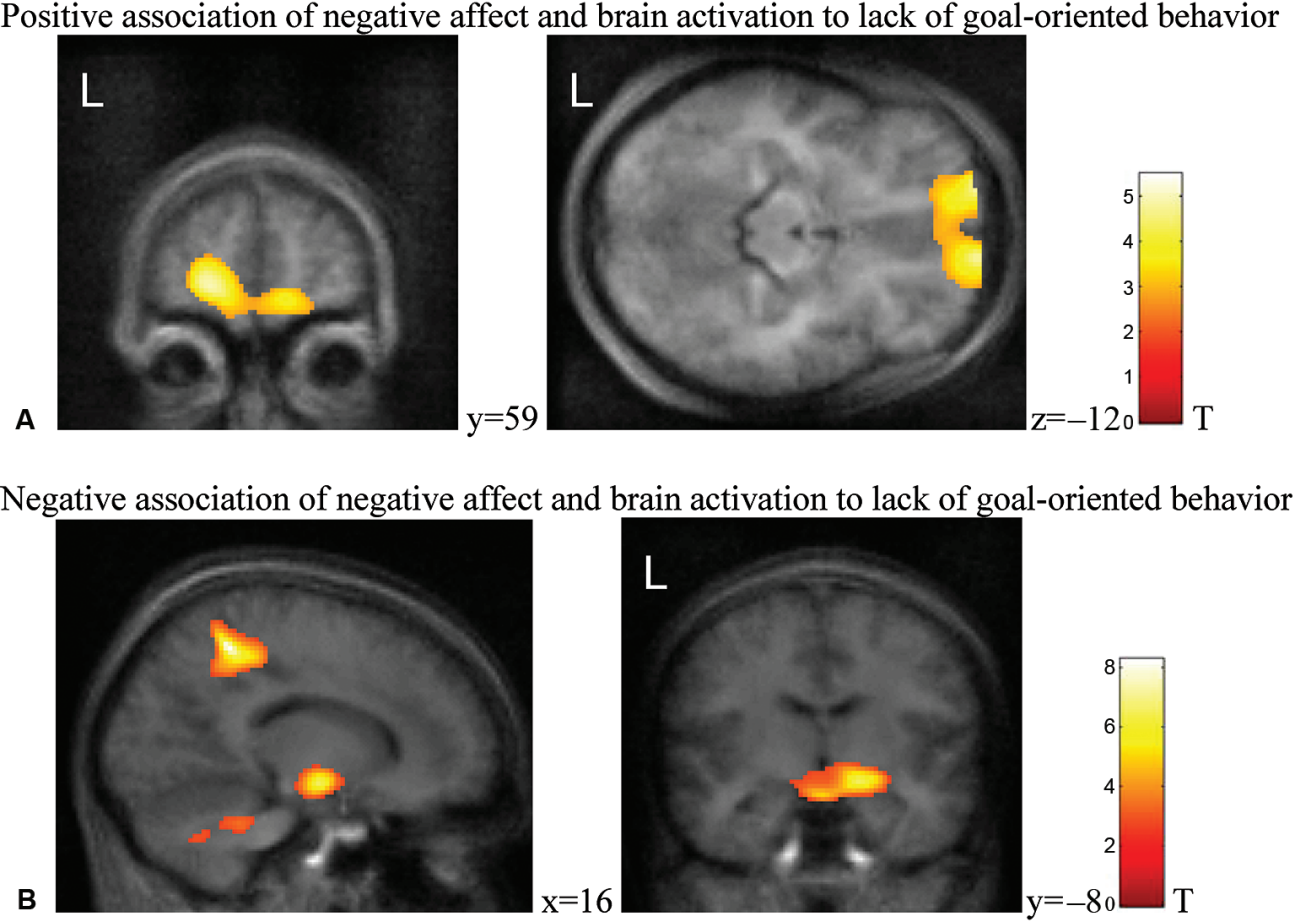
**Statistical maps of behavioral prediction of lower individual responsiveness to lack of goal-oriented behavior (threshold for cluster size according to *p* < 0.05 corrected)**. **(A)** Ventromedial prefrontal as well as **(B)** left precuneus and left hippocampal responses were associated with NA ratings. Positive association of NA and brain activation to lack of goal-oriented behavior. Negative association of NA and brain activation to lack of goal-oriented behavior.

Statistical mapping of the linear prediction of affect change correlating on the hemodynamic responses to low goal-directed behavior were calculated. PA correlated negatively with activation in bilateral insular cluster extending into the amygdala during phases low in goal-directed behavior (Figures 1A, B). Increase in NA was associated with activation in bilateral ventromedial prefrontal cortex (vmPFC) during phases without goal-oriented behavior (Figure 2A) and with right-lateralized deactivation in precuneus and hippocampus (Figure 2B; see Table 1 for the list of clusters associated with the boredom construct). The extent of the activation clusters yielded survival after correction for multiple comparisons across the volume. Peak *t*-values in contrast would not survive strict thresholds. This is in agreement with previous observation that subjective ratings are associated with rather distributed network activation or that the activation centers vary across individuals (Mathiak et al., 2011).

**Table 1:** **Cluster associated with boredom construct**.

Area	MNI coordinates			t-peak	k_E_	p
	x	y	z			
Reduced PA with low goal-directed behavior						
Insula R	50	12	2	5.34	2489	0.016
Insula L	−36	22	−22	4.91	3455	0.003
Increased NA with low goal-directed behavior						
vmPFC L and R	−26	70	−6	5.48	3370	0.004
Reduced NA with low goal-directed behavior						
Precuneus R	16	−48	50	8.26	2000	0.048
Hippocampus R	16	−8	−12	6.51	3351	0.004

*PA* *positive affect* *NA* *negative affect* *vmPFC* *ventromedial prefrontal cortex* *MNI* *Montreal Neurological Institute* *k_E_* *cluster size (voxels)* *R* *right* *L* *left* *p* *corrected *p*-value for cluster size.*

## Discussion

Virtual reality served as a model for complex social behavior, enabling recording of neural activity accompanying different affective outcomes. Considering boredom as affective outcome of prolonged phases with lowered goal-directed behavior during video game play, neural networks underlying affective control emerged. Increase of PA correlated with deactivation in amygdala and insula. Increase of NA, reflecting the dissatisfaction with the game experience, correlated with activation in vmPFC as well as deactivation of hippocampus and precuneus during prolonged inactive phases during game play.

Emotions elicited by or during the appraisal of external stimuli can be characterized according to different dimensions. In cognitive neuroscience, the most established concept differentiates valence and arousal. Basing on this model, Anders et al. (2004) demonstrated a functional segregation of brain structures underlying peripheral physiologic responses and verbal ratings along the emotional dimensions of valence and arousal. Valence of a stimulus as measured by startle responses correlated with amygdala activity whereas verbal reports of negative emotional valence were associated with insular activity. Further, peripheral physiological and verbal responses along the arousal dimension correlated with activity in thalamus and vmPFC. We adopted an alternative but widely accepted model, assuming that PA and NA dimensions are independent to a large extent (Huebner and Dew, 1996). Indeed, the experimental data support the existence of the separate neural circuits underlying the change of PA and NA and describe the approach system (facilitating appetitive behavior and generating certain types of PA that are approach-related) and withdrawal system (generating certain, withdrawal-related, forms of NA; for a review, see Davidson and Irwin, 1999). The PANAS measures both PA and NA and the correlation between the two scales is low and stable across different time frames (Watson, 1988). In agreement with these findings, changes of the two constructs were reflected in separate neural networks. NA depended on the activity of the vmPFC, putamen and hippocampus whereas PA correlated with activation of amygdala and insula. Similar to the study by Anders et al. (2004), the activation of the amygdala and insula correlated with one stimulus dimension and ventromedial prefrontal networks with the other one.

The amygdala and the PFC have extensive reciprocal connections and act together to regulate the processing of negative emotions. Diekhof et al. (2011) demonstrated that the vmPFC, accompanied by a concordant reduction of activation in the left amygdala, controlled negative affective responses. The cognitive reappraisal strategies were accompanied by a hyperactivation in the anterior cingulate and the insular cortex. Our study showed the dichotomy among those structures: while the vmPFC was involved in the processing of NA, the amygdala and insula were involved in processing of the PA.

Limbic structures with afferent functions such as amygdala and insula have been implicated in processing of negative emotions such as fear and disgust. Amygdala is a core structure involved in emotional processing, particularly of fear or anger (Dyck et al., 2011; see e.g., Costafreda et al., 2008; for a review). Consequently, increase of amygdala activation in individuals interfered with the experience of PA. In a similar vein, the anterior insula is suggested as being a central structure in mediating interoceptive awareness and the subjective experience of feelings through the representations of bodily reactions, consistent with the James—Lange theory of emotion and the somatic marker hypothesis (Craig, 2002, 2009; Damasio, 2003). It is believed to be responsible chiefly for negative emotions, in particular disgust (for a review, see Bossaerts, 2010). In line with this theory, an inhibition of the insula—similarly to the amygdala—may help to preserve the PA. Indeed, anterior insula may contribute to the mediation of fear-related arousal and negative affective states through its extensive reciprocal connections with the amygdala (Augustine, 1996; Anders et al., 2004). Alternatively, Sterzer and Kleinschmidt (2010) proposed that the anterior insula plays an integrative role in perception-action coupling. Driven by the salience of a sensory event, by task demands, or even by spontaneous activity fluctuations, insular activity mediates states of elevated sensory alertness and readiness for action. Derek (2011) considered a “boredom threshold” yielding reactivation of alternative perceptual concepts. This should render the individual more sensitive and more reactive to any kind of sensory information in situations that pose potential challenges to homeostasis. Accordingly, the game players who failed to decrease the insula activation adequately to lower task demands in the inactive game phases experienced lower PA.

The vmPFC controls emotion experience. This area receives inputs from sensory cortices and has extensive connections with emotional and affective areas including amygdala, striatum, and brainstem (Ridderinkhof et al., 2004) leading to hypotheses on its role in modulation of time course of emotional responding (Davidson, 1998). Further, the vmPFC is proposed to serve as an integrator of external and internal environment, capturing the emotional significance of events and coordinating the appropriate emotional response (for a review, see Barbas, 2000). The vmPFC may be directly involved in the representation of elementary positive and negative emotional states even in the absence of immediately present incentives (for a review, see Davidson and Irwin, 1999). Diekhof et al. (2011) demonstrated in their meta-analysis that the activation of the vmPFC reduced the degree of subjectively perceived unpleasantness. Contrary, the areas in medial and ventromedial PFC as well as subgenual anterior cingulate cortex activated in healthy participants during sad mood induction (Wang et al., 2006; Paulesu et al., 2010) and were hyperactive in patients with depression (Drevets et al., 2008). Moreover, an excitatory circuit within the vmPFC augmented fear expression, which is located dorsal to fear-inhibiting regions and could be capable of exciting the amygdala (Quirk and Beer, 2006). Diekhof et al. (2011) postulate the vmPFC as a controller of perceived fear and averseness that modulates negative affective responses in phylogenetically older structures of the emotion processing system, such as the amygdala. In this theoretical framework, the increased activation of vmPFC during prolonged inactivity in the game increased NA.

In our study, hippocampal activity seemed to counteract the experience of boredom. The hippocampus is the core structure involved in the formation and temporary storage of episodic and semantic memories, as well as in spatial navigation (for a review, see Stella et al., 2011). Similarly the precuneus was involved in reduction of boredom. A recent study using EEG source localization found a similar areal associated with the feeling of spatial presence during video games (Havranek et al., 2012). Presence in virtual environments (Baumgartner et al., 2008) is related to flow experience (Csimathkszentmihalyi, 2000; Faiola et al., 2013), which was found to be associated with precuneus activity as well (Klasen et al., 2011). In contrast to our study, the senso-motor network contributed flow (Klasen et al., 2011) and prefrontal networks to activity control in a first person simulation (Havranek et al., 2012), supporting a dissociation of boredom from these constructs. Episodic memory and engagement with the game may counteract subjective experience of boredom.

In a related account, the precuneus, along with adjacent areas within the posteromedial parietal cortex, contribute significantly to the “default mode” of brain function during conscious resting state (Cavanna, 2007). It is considered one of the core structures responsible for consciousness and self-representation (for a review, see Cavanna and Trimble, 2006). The precuneus may be involved in the generation of the spatial information necessary for imagined whole body movements and its activation preceding the beginning of imagined movement (Ogiso et al., 2000). Moreover, activation of precuneus was demonstrated in cognitive tasks requiring mental imagery, including visual rotation, deductive reasoning, music processing and mental navigation (Cavanna and Trimble, 2006). According to Watson et al. (1994), boredom is an externally driven state, the affective result of impoverished external stimuli, conceivably due to a lack of cognitive resources necessary to intrinsically generate interest. The activation of both precuneus and hippocampus may support the planning of coming actions during waiting periods and protect the game players from the feeling of boredom.

Despite the relatively clear findings concerning the neural networks, caution has to be taken with the generalization of the present study. Conceivably only aspects of boredom were assessed with this methodology. Boredom due to exhaustion such as fatigue cannot be considered in such short time scale nor can it be measured using fMRI. Indeed the considered change of affect as measured by the PANAS may not be sufficiently validated as a measure for mood effects. Therefore we also adhere to the label “change of affect” in reference to the PA and NA labels. A future challenge would be to establish a direct causal link between low activity and mood effects, which is only partially fulfilled in the current experiment.

Methodologically, the low number of participants must be considered an important limitation. In particular in a study with higher power, more networks contributing to boredom can be expected. The survival of clusters at the rather rigorous threshold with FWE-correction, however, indicates rather high effect sizes in the observed clusters. More seriously, the link between the affect measures and the lack of goal-directed behavior is only correlational. PANAS was conducted only directly before and after the fMRI measurements. Therefore additional events may have contributed to the changes in affect. Phases with lack of activity may have only been intercorrelated variables. This, however, is a general disadvantage of naturalistic studies. We studied rather unrestricted gaming behavior. Therefore the observed correlations cannot be directly interpreted as causal. Nevertheless the approach has the advantage that it reflects rather naturalistic behavior which is not hampered by intervening explicit mood assessments or experimental interventions. In future, alternative approaches should assess affect during the game play, e.g., by pop-up questions or peripheral physiological markers such as heart rate.

### Concluding remarks

We demonstrated neural contributions to boredom in video games. Conceivably, deactivation in putamen and hippocampus reflected decreased task-related mind-wandering and action planning while the increased activity in vmPFC were associated with the accompanying increase of NA and the decreased activity in amygdala and insula-improved PA. Moreover, our study confirmed that PA and NA are separate constructs, represented by distinct neural patterns, with vmPFC involvement in the processing of NA and the amygdala and insula in processing of PA. The results of our study shed new light on the mechanisms of emotional processing. Understanding better the concept of independent PA and NA as well as their neural correlates will improve our understanding of the emotional system in the brain.

## Conflict of interest statement

The authors declare that the research was conducted in the absence of any commercial or financial relationships that could be construed as a potential conflict of interest.
